# HDAC8 affects MGMT levels in glioblastoma cell lines *via* interaction with the proteasome receptor ADRM1

**DOI:** 10.18632/genesandcancer.197

**Published:** 2019

**Authors:** Irene Santos-Barriopedro, Yixuan Li, Sonali Bahl, Edward Seto

**Affiliations:** ^1^ George Washington Cancer Center, Department of Biochemistry & Molecular Medicine, George Washington University School of Medicine & Health Sciences, Washington, DC, USA

**Keywords:** HDAC, DNA damage, cell cycle, temozolomide, drug resistance

## Abstract

Temozolomide (TMZ) is an alkylating agent chemotherapy drug used as a first-line treatment for glioblastoma multiforme (GBM). O^6^-methyl-guanine DNA methyltransferase (MGMT) repairs DNA damage induced by TMZ; hence, elevated MGMT levels usually correlate with TMZ resistance. MGMT promoter methylation is a key regulatory mechanism for MGMT expression and is important in overcoming TMZ therapy resistance. To date, little is known about how MGMT expression is regulated beyond promoter methylation. In this work, we show an alternative mechanism by which MGMT levels are regulated independent of its promoter methylation status. We found that inhibition of the histone deacetylase HDAC8 by either HDAC8-specific inhibitor PCI34051 or HDAC8 shRNA decreases MGMT levels in GBM cell lines. Furthermore, the proteasome receptor ADRM1 participates in this MGMT regulation by interacting with HDAC8. Interestingly, this interaction is disrupted by TMZ exclusively in TMZ sensitive cells, suggesting that this MGMT regulatory pathway might be inactivated in TMZ resistant cells. Consequently, HDAC8 inhibition in GBM cell lines increases DNA damage and cell cycle arrest and, eventually, decreases cell viability, likely due to the decrease in MGMT protein levels.

## INTRODUCTION

Glioblastoma multiforme (GBM) is the most common and lethal primary brain tumor in adults. The therapeutic approach that is often used for this cancer is surgical resection followed by radiotherapy and chemotherapy with temozolomide (TMZ), with the average post-treatment survival time of 12-15 months [1]. The number of drugs used to treat GBM is limited due to the presence of the blood-brain barrier, and no treatments are curative. So far, TMZ is one of the most suitable drugs for this cancer as its lipophilic nature allows the molecule to cross the blood-brain barrier. Moreover, TMZ is activated in an alkaline environment, which is present in GBM but is not found in normal cells [2]. TMZ is an alkylating reagent that induces DNA damage, resulting in the activation of DNA repair machineries [3,4]. The main mechanism of action of TMZ is the transfer of a methyl group to guanine to generate O^6^-methylguanine-DNA, which can be repaired by the O^6^-methylguanine-methyltranferase DNA repair enzyme (MGMT). MGMT transfers the methyl group from the guanine to its own cysteine. Massive DNA damage induction by TMZ entails a deficient DNA repair by MGMT and leads to the activation of the mismatch repair system. Importantly, elevated MGMT levels are correlated with poor prognosis in cancer. MGMT transcription is regulated by different factors, such as transcription factors (Sp1, NF-κB, CEBP and AP-1), microRNAs and gene promoter methylation [5]. Aberrant MGMT transcriptional regulation in GBM increases MGMT levels in those tumors. Targeting MGMT transcriptional regulation with consideration of promoter methylation has become a priority in GBM treatment.

Post-translational modifications are important for the regulation of cell physiology and are involved in tumorigenic processes. Acetylation is one type of post-translational modification that can occur on histones [6] and is controlled by histone acetyltransferases (HATs) and histone deacetylases (HDACs), which transfer and remove acetyl groups from proteins, respectively [7]. HDACs are generally considered repressors of gene expression because of their effects on histones. However, deacetylation can also modify non-histone proteins, which in turn regulate the function of those proteins. There are 18 different HDACs organized in four classes based on their structural homology (I, II, III and IV). They harbor a conserved catalytic site, facilitating the development of non-selective HDAC inhibitors (HDACis) that have been used in cancer therapies [8]. HDACis can also be used for the treatment of GBM and are in clinical trials to test their combination with other drugs [9]. HDACis affect cell proliferation, angiogenesis, cell invasion and migration in GBM. Additionally, they reduce the number of GBM cancer stem cells [10] and activate natural killer cells in the immune system to target tumorigenic cells [11]. Also, specific HDACs are currently under more extensive study, as inhibiting one particular HDAC may result in better clinical outcomes and less side effects. For example, inhibition of both HDAC4 and HDAC6 in GBM induces DNA damage [12]. Furthermore, HDAC2 downregulation in GBM results in less proliferation and confers increased sensitivity to TMZ and greater cell motility [13]. However, it is difficult to design an inhibitor specific for these HDACs that can cross the blood-brain barrier.

HDAC8 is a class I HDAC that is ubiquitously expressed but is particularly abundant in the brain, prostate and kidney [14]. It is involved in neuron differentiation [15] and is essential for the development of the skull [16]. Unlike many other HDACs, HDAC8 does not require other factors to perform its activity and harbors both deacetylase and deacylase activity, removing acetyl and long fatty acid chains from its substrates, respectively [17]. The best-characterized HDAC8 substrate is the cohesin SMC3. Mutation of SMC3 or HDAC8 is implicated in the development of Cornelia de Lange Syndrome [18]. Several studies confirmed that HDAC8 acts as an oncogene in different tumors, including gastric cancer, neuroblastoma, T-cell lymphocytes, hepatocarcinoma and breast cancer. As an example, HDAC8 downregulation increases the sensitivity to doxorubicin therapy in neuroblastoma [19] and induces the differentiation of malignant cells into neurons [19–21]. However, HDAC8 has not been extensively studied in many other processes.

Here we report for the first time a link between HDAC8 and GBM. We found that HDAC8 regulates MGMT protein levels via its interaction with the proteasome receptor ADRM1. TMZ treatment causes the dissociation of HDAC8 from ADRM1 in TMZ-sensitive U87 glioblastoma cells. However, the HDAC8 and ADRM1 interaction cannot be disrupted by TMZ treatment in TMZ-resistant T98G cells. Our results suggest that HDAC8 inhibition increases DNA damage, triggering cell cycle arrest and affecting the GBM cell viability, likely due, in part, to the decrease in MGMT levels.

## RESULTS

### HDAC8 inhibition affects cell viability in glioblastoma cell lines

GBM relapses and acquires resistance to therapies, including the commonly used TMZ. Previous characterization of GBM cell lines indicates that altered molecular mechanisms are involved in maintaining TMZ resistance and sensitivity [22]. In our study, we used the well-characterized TMZ-sensitive U87 and TMZ-resistant T98G glioblastoma cell lines (Supplementary Figure 1A). T98G cells are resistant to TMZ mainly due to elevated MGMT levels and the base excision repair enzyme, alkylpurine-DNA-N-glycosylase (Supplementary Figure 1B) [22].

**Figure 1:**
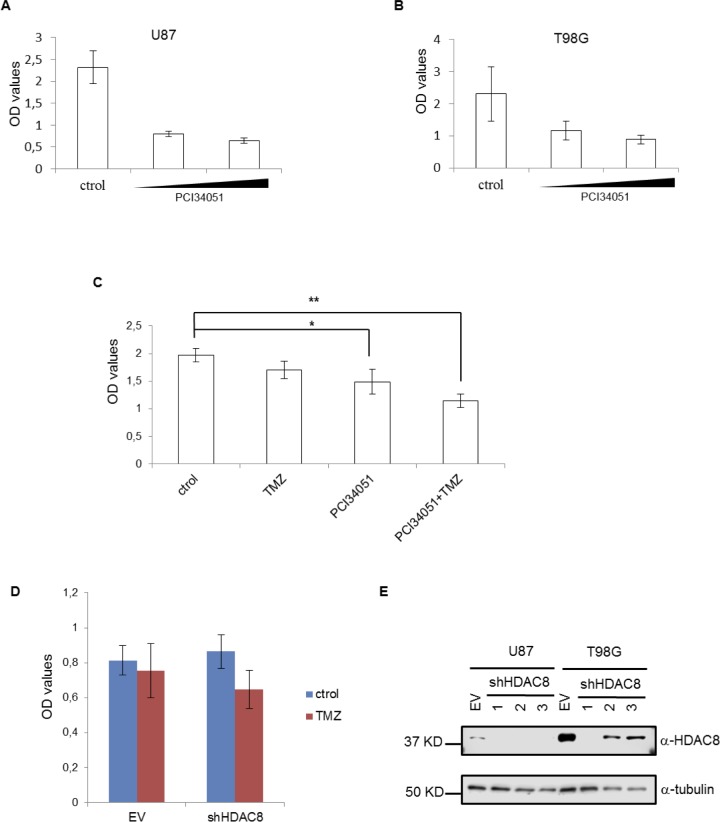
PCI34051 decreases U87 and T98G viability **A.** and **B.** Cell viability determined by CCK-8 assay after treatment with 30 and 40 µM PCI34051 for 4 days. **C.** T98G cell viability determined with the CCK-8 assay after 3 days’ treatment with either 30 µM PCI34051 or 250 µM TMZ. *n* = 3 *p < 0.05, **p < 0.005. **D.** T98G cell (Empty vector or TRCN0000350469 shHDAC8) viability determined with the CCK-8 assay after 4 days’ treatment with 250 µM TMZ. **E.** Extracts of U87 and T98G cells expressing empty vector (EV) or shHDAC8 (1- TRCN0000350469, 2-TRCN0000004852 and 3-TRCN0000314874) were subject to Western blotting with tubulin and HDAC8 antibodies.

As mentioned above, HDAC8 is known to be involved in initiation and progression of different cancers; however, the role of HDAC8 in GBM remains unexplored. In this work, we used the well-known and commercially available HDAC8-specific inhibitor PCI34051 [23] to observe its effect on GBM cell lines. Cells were treated with increasing concentrations of PCI34051, and effects of the inhibitor, such as viability and phenotype, were observed in both cell lines in a dose-dependent manner (Supplementary Figure 2). Cell proliferation decreased following PCI34051 treatment, suggesting that HDAC8 is required for the viability of GBM cell lines (Figure 1A and 1B). Combination of TMZ with either PCI34051 or HDAC8 shRNA decreases T98G viability (Figure 1C and 1D). Additionally, HDAC8 protein levels are more elevated in T98G cells than in U87 cells (Figure 1E), suggesting that HDAC8 could be another factor of resistance against TMZ.

### HDAC8 regulates MGMT protein levels

**Figure 2:**
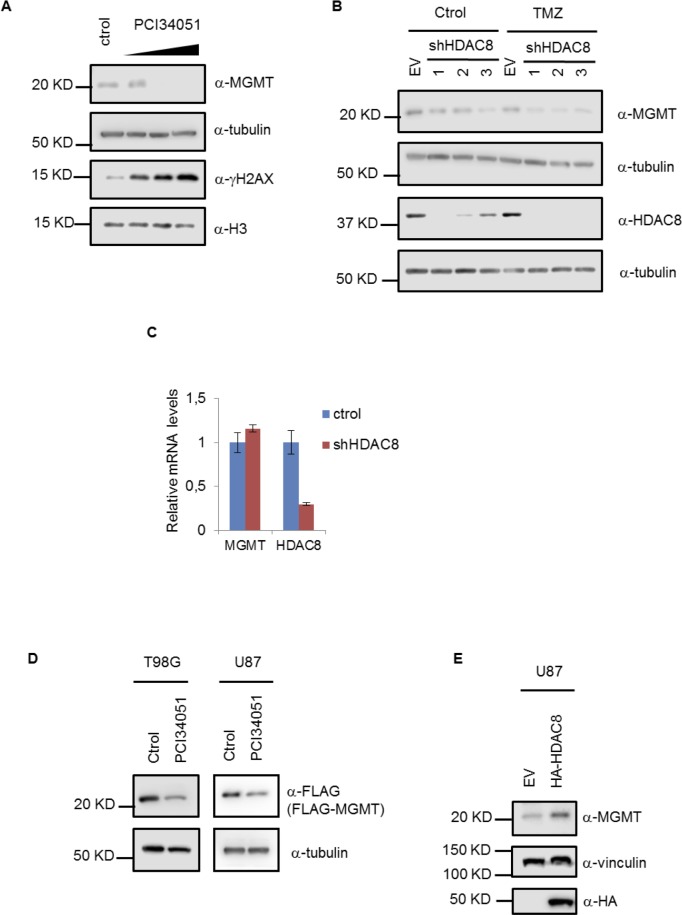
HDAC8 regulates MGMT protein levels **A.** Extracts from T98G cells treated with 15, 20 and 30 µM PCI34051 for 24 hours were subject to Western blotting with MGMT, tubulin, γH2AX and H3 antibodies. **B.** Extracts from T98G cells expressing stable shHDAC8 (1- TRCN0000350469, 2-TRCN0000004852 and 3-TRCN0000314874) and treated with TMZ for 48 hours were subject to Western blotting with MGMT, tubulin and HDAC8 antibodies. **C.** Quantitative RT-PCR analysis of mRNA from T98G cells expressing stable shHDAC8 (TRCN0000350469). **D.** Extracts from T98G and U87 cells expressing stable FLAG-MGMT and treated with PCI34051 for 24 hours were subject to Western blotting with FLAG and tubulin antibodies. **E.** Extracts from U87 cells were subject to Western blotting with MGMT, vinculin and HA antibodies.

Elevated MGMT levels confer resistance to GBM against TMZ. The elevation of MGMT levels has been rationalized as the effect of an alteration in transcription regulation due to the dysregulation of different transcription factors, DNA methylation in the promoter or microRNAs [5]. T98G cells are characterized by high MGMT levels. We found that PCI34051 treatment decreases MGMT levels in T98G cells, correlating with an increase in phosphorylated H2AX (γH2AX) levels, a DNA damage marker, suggesting that the reduction in MGMT levels increases DNA damage in this cell line (Figure 2A). However, the HDAC8 inhibitor may induce other side effects that are unrelated to HDAC8 activity. In order to attribute this effect to the inhibition of HDAC8 activity, we used HDAC8-specific shRNA to deplete HDAC8 in T98G cells. HDAC8 KD cell lines show a decrease in MGMT protein levels compared to the control (Figure 2B), confirming the result observed after PCI34051 treatment. No changes in MGMT levels in the control cells vs TMZ treated cells were observed, as described before [24].

The effects observed in MGMT levels could be due to changes at the transcriptional level. However, MGMT mRNA levels remain unaffected in HDAC8 KD cell lines, suggesting that MGMT transcription is not regulated by HDAC8 (Figure 2C). To further confirm that HDAC8 could regulate MGMT expression at the post-transcriptional level, we generated a stable cell line expressing FLAG-tagged MGMT in both U87 and T98G. In this case, exogenous FLAG-MGMT is expressed under a CMV promoter; consequently, it is constitutively expressed regardless of the regulation of the endogenous MGMT. We found that both PCI34051 (Figure 2D) and HDAC8 KD (Figure 4C) decrease exogenous MGMT levels. Moreover, we observed that HDAC8 overexpression increases endogenous MGMT levels in U87 cells (Figure 2E); however, ectopic HDAC8 cannot further upregulate MGMT in T98G cells (data not shown), which might be because the elevated MGMT level is already saturated in TMZ resistant cells.

### HDAC8 interacts with ADRM1 to regulate MGMT levels

**Figure 3:**
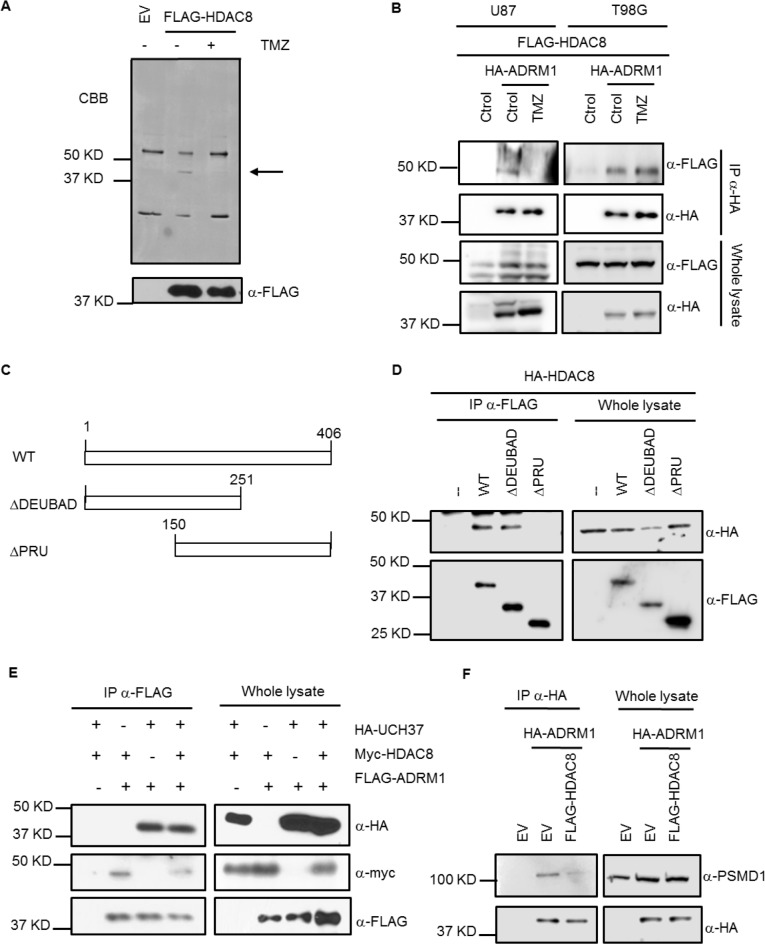
HDAC8 binds to ADRM1 through the PRU domain **A.** Extracts from U87 cells treated with TMZ for 48 hours were immunoprecipitated with a FLAG antibody and subject to Coomassie blue staining. **B.** HA immunoprecipitation of extracts from U87 and T98G cells expressing FLAG-HDAC8 and HA-ADRM1 and treated with TMZ for 48 hours. **C.** Schematics of the different FLAG-ADRM1 constructs used in D. **D.** FLAG immunoprecipitation of extracts from HeLa cells transfected with different constructs of FLAG-ADRM1 and HA-HDAC8. **E.** FLAG immunoprecipitation of extracts from HeLa cells transfected with HA-UCH37, myc-HDAC8 and FLAG-ADRM1. **F.** HA immunoprecipitation of extracts from T98G cells expressing HA-ADRM1 and FLAG-HDAC8.

TMZ treatment triggers the activation of different types of DNA repair, resulting in different downstream effects, such as cell cycle arrest and apoptosis due to the activation of different DNA repair machineries [2]. MGMT repairs DNA damage induced by TMZ. Given that HDAC8 regulates MGMT protein levels, it is possible that TMZ may affect HDAC8. We found that HDAC8 levels and localization are unaffected by TMZ (Supplementary Figures 3A and 3B). Surprisingly, we found that TMZ treatment changes the interaction between HDAC8 and a protein of approximately 45 KDa, by promoting their dissociation (Figure 3A and Supplementary Figure 4). Mass spectrometry analysis allowed the identification of the proteasome receptor ADRM1 (also called Rpn13) as the interacting protein. We confirmed that ADRM1 and HDAC8 interact, and that this interaction can be disrupted by TMZ in the TMZ-sensitive U87 cell line, but not in the TMZ-resistant T98G cell line (Figure 3B and Supplementary Figure 5). Surprisingly, this interaction remains unaffected by TMZ in T98G cells, suggesting that this mechanism might be involved in the resistance to TMZ apart from that already described for this cell line [22].

ADRM1 contains two different domains: the N-terminal pleckstrin-like receptor of ubiquitin (PRU) domain and the DEUBiquitinase ADaptor (DEUBAD) domain. The deubiquitinase UCH37 binds to ADRM1 through its DEUBAD domain at the proteasome. ADRM1 requires the interaction through its PRU domain of both the proteasome subunit PSMD1 and the ubiquitinated substrate in order to function as a proteasome receptor [25]. We subcloned truncated forms of ADRM1 (Figure 3C): ΔDEUBAD (without the C-terminal DEUBAD domain) and ΔPRU (without the N-terminal PRU domain) in order to determine the domain that interacts with HDAC8. We found that HDAC8 binds to ADRM1 through the PRU domain (Figure 3D). This interaction does not affect the interaction between UCH37 and ADRM1 (Figure 3E). However, it appears that HDAC8 overexpression inhibits the interaction between ADRM1 and PSMD1 (Figure 3F), suggesting that the interaction of HDAC8 with ADRM1 excludes the interaction between ADRM1 and the proteasome.

**Figure 4:**
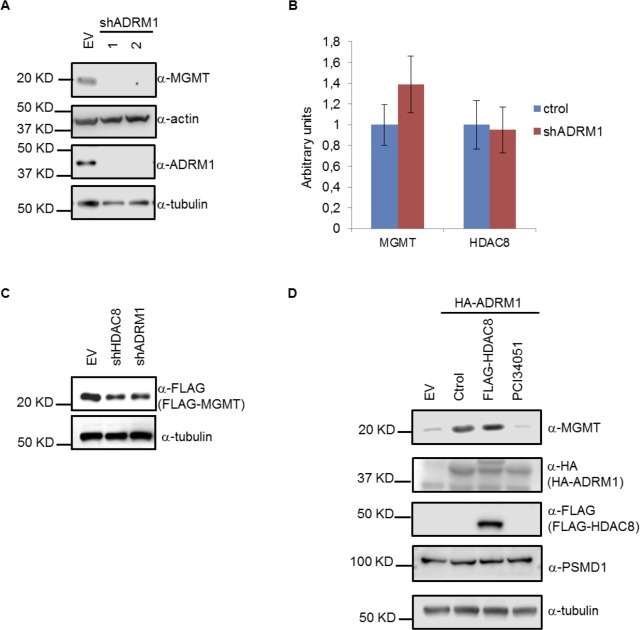
ADRM1 regulates MGMT protein levels through HDAC8 **A.** Extracts from T98G cells expressing shADRM1 (1-TRCN0000286432, 2-TRCN0000293817) were subject to Western blotting with MGMT, actin, tubulin and ADRM1 antibodies. **B.** Quantitative RT-PCR analysis of mRNA from T98G cells stably expressing shADRM1 (TRCN0000286432). **C.** Extracts from T98G cells expressing FLAG-MGMT, shHDAC8 (TRCN0000350469) and shADRM1 (TRCN0000286432) were subject to Western blotting with FLAG and tubulin antibodies. **D.** Extracts from T98G cells either expressing HA-ADRM1, FLAG-HDAC8 or treated with 15 µM PCI34051 for 24 hours were subject to Western blotting with MGMT, HA, FLAG, PSMD1 and tubulin antibodies.

We generated a stable cell line with shADRM1 in order to obtain the ADRM1 KD T98G cell line (Figure 4A). Unexpectedly, MGMT protein levels decrease in ADRM1 KD cells (Figure 4A). Moreover, MGMT mRNA levels remain unchanged in ADRM1 KD cells (Figure 4B), as was observed with HDAC8 KD. Next, we found in the exogenous FLAG-MGMT cell line that exogenous MGMT is also regulated by ADRM1 shRNA in the same proportion as by HDAC8 shRNA (Figure 4C). We found that ADRM1 overexpression increases MGMT levels, and that PCI34051 eliminates this effect (Figure 4D), indicating that the effect of ADRM1 on MGMT depends on HDAC8. Altogether, these results suggest that the association of HDAC8 and ADRM1 might contribute to the high levels of MGMT in TMZ-resistant cells. In U87 TMZ-sensitive cells, the interaction between HDAC8 and ADRM1 can be disrupted by TMZ treatment; however, this association remains unaffected upon TMZ treatment in resistant T98G cells, which causes the upregulation of MGMT in T98G cells and further confers the TMZ-resistant phenotype.

### HDAC8 inhibition induces DNA damage and cell cycle arrest

**Figure 5:**
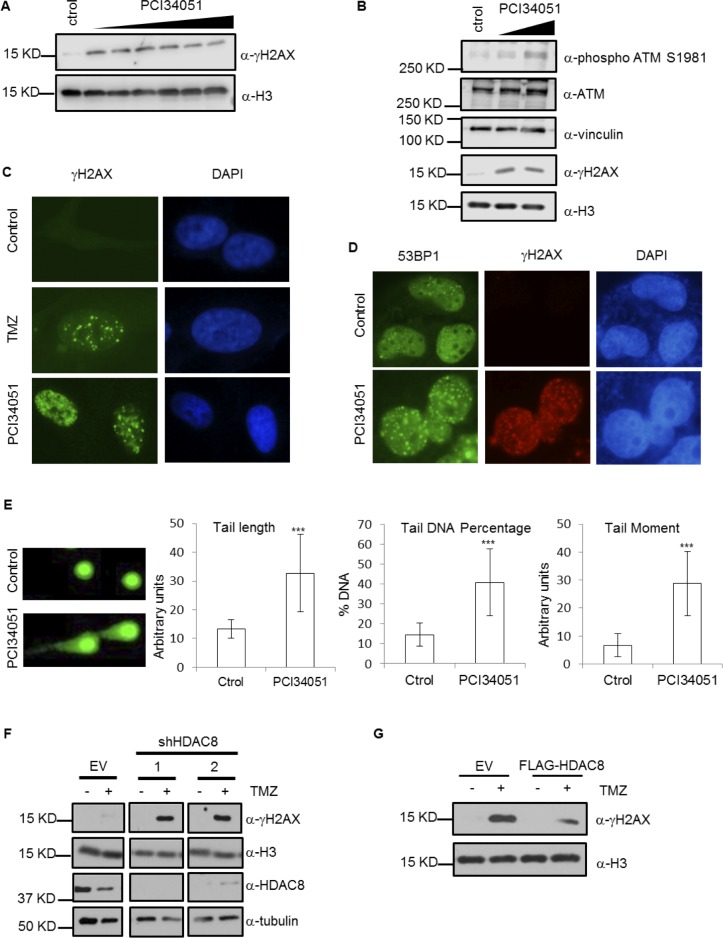
HDAC8 inhibition induces DNA damage **A.** Extracts from U87 cells treated with PCI34051 for 24 hours were subject to Western blotting with γH2AX and H3 antibodies. **B.** Extracts from T98G cells treated with 15 µM PCI34051 for 24 hours were subject to Western blotting with phosphorylated ATM S1981, ATM, vinculin, γH2AX and H3 antibodies. **C.** Immunofluorescence demonstrating γH2AX expression in U87 cells treated with TMZ and 15 µM PCI34051 for 24 hours. **D.** Immunofluorescence showing 53BP1 and γH2AX expression in T98G cells treated with PCI34051 for 24 hours. **E.** Alkaline comet assay analysis of T98G cells treated with 20, 30 and 40 µM PCI34051 for 24 hours, *n* > 20, ***p < 0.0005. **F.** Extracts from U87 cells expressing shHDAC8 (1- TRCN0000350469, 2-TRCN0000004852) and treated with TMZ for 48 hours were subject to Western blotting with γH2AX, H3, HDAC8 and tubulin antibodies. **G.** Extracts of U87 cells expressing FLAG-HDAC8 and treated with TMZ for 48 hours were subject to Western blotting with γH2AX and H3 antibodies.

Aberrant activation of different DNA repair pathways (defective mismatch repair, hyperactivated base excision repair or elevated MGMT levels) confers resistance against TMZ treatment [26]. Based on our observation that HDAC8 affects MGMT levels, we expected that DNA damage would be affected by HDAC8 inhibition. We checked whether PCI34051 induces DNA damage by measuring γH2AX and phosphorylated ATM levels. The results show that PCI34051 increases the levels of these markers (Figure 5A and 5B) and additionally increases the number of γH2AX foci (Figure 5C). In addition, the number of 53BP1 foci in the nucleus is increased under PCI34051 treatment (Figure 5D). For a final confirmation, we performed the alkaline comet assay (Figure 5E) and found that different parameters, such as tail length, tail DNA percentage and tail moment, are increased upon PCI34051 treatment. To confirm the participation of HDAC8 in DNA damage, we assessed DNA damage levels in HDAC8 KD cells after TMZ treatment and found that HDAC8 downregulation enhances γH2AX levels in response to TMZ treatment (Figure 5F), whereas HDAC8 overexpression decreases γH2AX levels (Figure 5G). These results suggest that HDAC8 depletion increases DNA damage, as expected, correlating with the MGMT level decrease.

**Figure 6:**
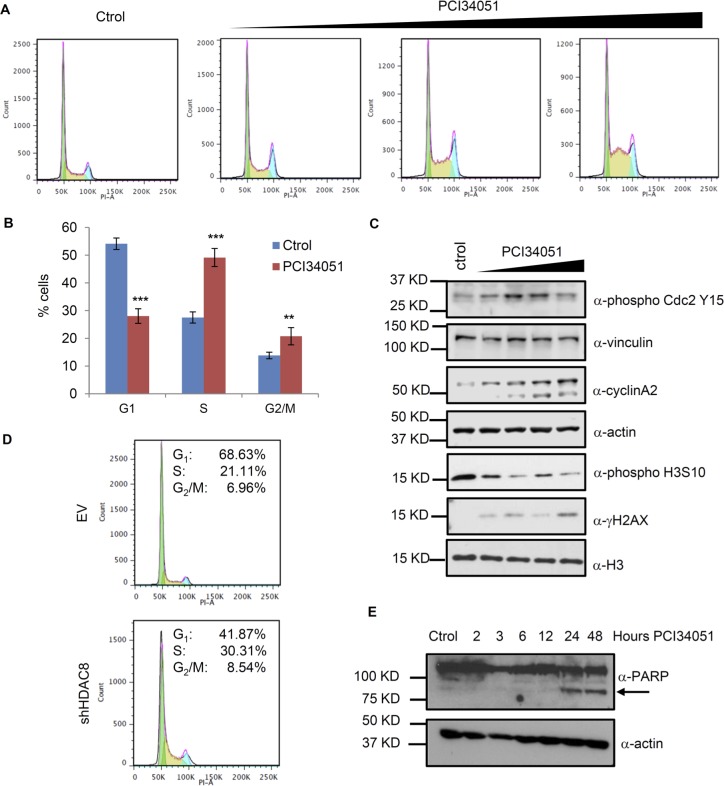
HDAC8 inhibition induces cell cycle arrest **A.** Flow cytometry analysis of T98G cells treated with 15 µM PCI34051 for 24 hours and stained with propidium iodide. **B.** Quantitation of the percentage of cell lines in the different phases from the flow cytometry analysis, *n* = 20000, **p < 0.005, ***p < 0.0005. **C.** Extracts of T98G cells were subject to Western blotting with phosphorylated Cdc Y15, vinculin, Cyclin A2, actin, phosphorylated H3 S10, γH2AX and H3 antibodies. **D.** Flow cytometry analysis of T98G cells expressing shHDAC8 (TRCN0000350469) and stained with propidium iodide. **E.** Extracts of U87 cells treated with PCI34051 were subject to Western blotting with PARP and actin antibodies.

Massive DNA damage induces the activation of checkpoints and, eventually, cell cycle arrest. Accumulation of the O^6^-methylguanine DNA lesions induces arrest in S-phase due to the participation of mismatch repair [4, 26]. TMZ affects cell cycle progression, increasing the number of cells in late S and G_2_/M phases in cells with low MGMT levels [27, 28]. HDAC8 inhibition increases DNA damage drastically and affects the proliferation and viability of the cells (Figure 1A and 1B); therefore, HDAC8 inhibition may additionally affect cell cycle progression. We analyzed the cell cycle in T98G asynchronous cells after treatment with PCI34051 and found that cells are arrested at both S-phase and G_2_/M-phase (Figure 6A and 6B). To verify that HDAC8 inhibition affects the cell cycle, we evaluated the levels of several cell cycle-related genes and modifications (Figure 6C). We found that phosphorylated cdc2 and Cyclin A levels are increased upon HDAC8 inhibition, indicating that there is an arrest in G_2_/M phase that mimics the same effect as described with TMZ treatment [29]. We found that phosphorylated H3S10 levels decreased, indicating that cells mostly failed to enter mitosis. Next, we used HDAC8 KD cells to study the cell cycle and confirm that a decrease in HDAC8 levels leads to a higher cell percentage in S- and G_2_/M –phase, as observed with the HDAC8 inhibitor (Figure 6D).

A decrease in cell viability is often linked to an increase in apoptosis. The increase in cleaved PARP after PCI34051 treatment (Figure 6E) indicates that the cells might be undergoing apoptosis. We also found that PCI34051 induces autophagy, based on the accumulation of LC3 foci and increase in LC3 II, a marker of the autophagy process (Supplementary Figure 6A and 6B). Overall, these changes in cell phenotype demonstrate the effects of HDAC8 inhibition in U87 and T98G cell lines.

## DISCUSSION

**Figure 7:**
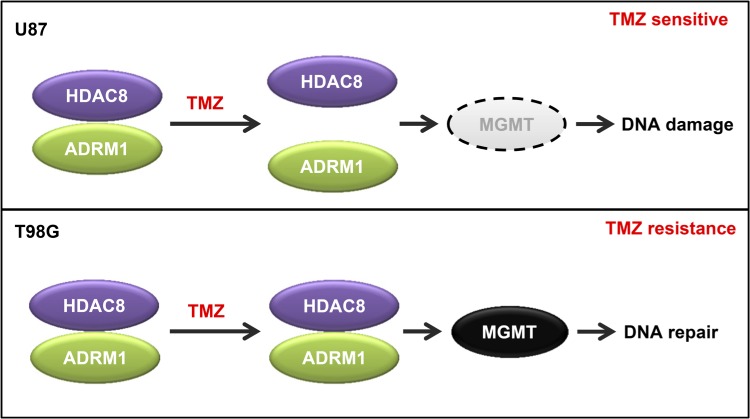
Proposed model for the regulation of MGMT by HDAC8 and ADRM1 HDAC8 regulates MGMT protein levels via its interaction with ADRM1. In TMZ-sensitive U87 cells, TMZ treatment causes the dissociation of HDAC8 from ADRM1, which results in the downregulation of MGMT together with accumulated DNA damage in cells. In TMZ-resistant T98G cells, the HDAC8 and ADRM1 interaction cannot be disrupted by TMZ treatment. The high level of MGMT expression confers resistance against TMZ treatment.

High MGMT expression confers resistance to GBM cell lines against TMZ treatment. This study shows that HDAC8 inhibition induces a decrease in MGMT protein levels. We found that HDAC8 binds to ADRM1 and TMZ treatment dissociates the HDAC8-ADRM1 interaction in TMZ-sensitive cells but not in resistant cells (Figure 7). We confirmed that ADRM1 also regulates MGMT protein levels, and our results indicate ADRM1 and HDAC8 interact to regulate MGMT levels. Through regulating the MGMT DNA repair protein, HDAC8 inhibition increases DNA damage, promotes cell cycle arrest and decreases cell viability.

HDACis such as vorinostat are FDA approved anticancer drugs [8]. For improved HDACi therapy in solid tumors, broad spectrum HDAC inhibitors are usually combined with other drugs that target key biological pathways, such as different DNA repair pathways [30]. However, HDAC inhibitors currently in use for cancer treatments are nonselective and target multiple HDACs, resulting in unwanted side effects, such as ventricular arrhythmia, thrombocytopenia or fatigue. Furthermore, several HDACs have both redundant and specific functions in the organism [7]; therefore, the application of non-specific HDAC inhibitors may not be effective as an anticancer therapy. Deciphering the function of each individual HDAC, as well as developing specific inhibitors is critical to better target certain tumors and reduce side effects.

The development of specific HDACis has many challenges due to the high resemblance in the catalytic mechanism and active site among all of the HDAC family members. HDAC8 possesses a unique flexible L1 loop at the N-terminal region of the protein that accommodates HDAC8 substrates [31]. This domain is missing in other HDACs and its presence has facilitated the development of highly specific HDAC8 inhibitors [32]. Therefore, the study of HDAC8 function in tumorigenic processes is important because the chance of generating a specific inhibitor is greater for HDAC8 than for most other classical HDACs. Here, we provide insights into HDAC8 function in GBM. The main obstacle for the development of therapies targeting GBM is the blood-brain barrier that the drugs must cross. The effect of HDAC8 inhibition in GBM cell viability and the possibility of developing specific HDAC8 inhibitors provide an opportunity to develop an HDAC8 inhibitor that can cross the blood-brain barrier and target GBM. Furthermore, the hypothetical HDAC8 inhibitor could overcome TMZ resistance in GBM.

HDAC8 can deacetylate different proteins in order to regulate their functions; however, HDAC8 deacetylation kinetics measured *in vitro* is lower compared to other Class I HDACs. Surprisingly, HDAC8 is more efficient in removing long fatty chains rather than acetyl groups from proteins [17]. We tested whether ADRM1, UCH37 or MGMT are deacetylated by HDAC8, but no changes in acetylation levels were detected (data not shown). We wonder whether, in this case, HDAC8 is deacylating, instead of deacetylating, these potential substrates. In either case, the identification of HDAC8 substrates and the amino acid involved in this process would be interesting and a prerequisite to obtaining a complete understanding of how HDAC8 might be targeted for treatment of GBM.

HDAC8 downregulation enhances TMZ-induced DNA damage, probably due to a decrease in MGMT levels. Downregulation of MGMT levels does not increase DNA damage [33]; however, in our study, only HDAC8 inhibition is able to induce DNA damage without the presence of additional stress. One possibility is that HDAC8 affects both MGMT levels and other pathways that are inducing DNA damage. Alternatively, it is possible that PCI34051 may have other side effects related to DNA damage. Interestingly, HDAC8 levels are higher in T98G cells than in U87 cells, indicating that HDAC8 might constitute a marker for resistance, therefore becoming a target to overcome TMZ resistance.

In this study we observed that HDAC8 inhibition arrests cells in S-phase and G_2_/M phase. It is likely that DNA damage caused by HDAC8 inhibition induces this arrest. It has been reported that HDAC8 inhibition affects the cell cycle in some cell lines, such as MCF7 [34, 35], an effect that is attributed to its deacetylase activity on the cohesin SMC3; however, in HeLa cells this effect is absent [18]. It is possible that HDAC8 induces cell cycle arrest in these cell lines because it affects DNA damage as well. Alternatively, the effect of HDAC8 on ADRM1 affects cell cycle progression because ADRM1 inhibition affects the cell cycle and blocks DNA replication and G2 arrest [36].

TMZ adds methyl groups to the N^7^ and O^6^ positions on guanine and N^3^ position on adenine that need to be removed to prevent DNA damage. The main DNA repair mechanisms involved in the removal of these methyl groups are mismatch repair, base excision repair and MGMT [22, 37]. Eventually, some cells develop resistance to TMZ because they have mechanisms to bypass the checkpoints or they possess aberrant DNA repair mechanisms; the best alternative to overcome this issue would be to combine TMZ treatment with other drugs that target different DNA repair pathways [37]. Overall, TMZ causes cell stress and it is not surprising to observe other effects, such as DNA hypermethylation, arising from this drug treatment [28]. We observed that the interaction between ADRM1 and HDAC8 is disrupted by TMZ as an example of another effect produced by TMZ treatment; however, the mechanism causing this dissociation remains unknown. This interaction is not disturbed by TMZ in TMZ-resistant cells, however, suggesting that the mechanism has been altered in those resistant cells. It would be interesting to decipher the mechanism that induces this dissociation and compare it with the resistant cells.

It has been proven that elevated MGMT levels correlate with the epigenetic regulation of its promoter. Particularly, the unmethylated DNA at the MGMT promoter correlates with high MGMT expression and is often found as a marker for the resistance against TMZ therapy [5]. In our study, HDAC8 and ADRM1 affect the MGMT protein levels instead of directly regulating the transcription of MGMT through its promoter.

ADRM1 is a proteasome receptor that binds to K48-linked diubiquitin in different polyubiquitinated proteins [38]. Furthermore, ADRM1 activates the deubiquitinase UCH37 and avoids its auto-inhibition. Prior to proteasome degradation, UCH37 removes the ubiquitin chains from those proteins [39]. Few proteins are known to be recognized by ADRM1 and the global ubiquitination levels remain unaffected after ADRM1 depletion. Surprisingly, in GBM, ADRM1 function favors MGMT stability instead of promoting its degradation. HDAC8 interaction does not affect the UCH37-ADRM1 interaction. However, in this case, HDAC8 affects the interaction between ADRM1 and the proteasome. These results suggest that ADRM1 has additional distinct functions that have yet to be described. ADRM1's affinity to ubiquitinated substrates is drastically decreased when it does not bind to the proteasome because the DEUBAD and PRU domains bind each other and are unable to recognize the ubiquitinated substrate [40]. Conceivably, HDAC8 may block ADRM1 function as a proteasome receptor towards the recognition of MGMT as a substrate to be degraded. However, this hypothesis does not fit with our observation that MGMT levels decrease after ADRM1 downregulation. It could be that ADRM1 affects the protein levels of another protein that regulates MGMT levels. Further research is required in order to elucidate how MGMT levels are controlled by ADRM1.

ADRM1 is overexpressed in multiple myeloma, ovarian cancer, colon cancer and gastric cancer [25]. Two ADRM1 inhibitors have been developed: RA190 and KDT-11. ADRM1 inhibition through the inhibitor RA190 induces apoptosis in multiple myeloma cell lines, suggesting that it might be a good target for multiple myeloma treatment [41]. Although the role of ADRM1 in GBM has not been reported, there is a link between ADRM1 and neurons due to its capacity to affect autophagy, a process that is required for the maintenance of those cell types to avoid the accumulation of useless proteins [25]. We found that HDAC8 inhibition induces autophagy in GBM cell lines and the finding that HDAC8 binds to ADRM1 indicates that ADRM1 might regulate autophagy in this process. However, in our study, the observable effects on autophagy may be due to the decrease in MGMT levels and, therefore, the induction of DNA damage. On the other hand, UCH37, the deubiquitinase activated by ADRM1, inhibits glioma cell migration and invasion [42], suggesting that ADRM1 inhibition could have a positive or negative effect on tumor progression, depending on the stage of the tumor.

The discovery of a new role for HDAC8 and ADRM1 in MGMT regulation expands the possibilities of the development of new therapies to overcome TMZ resistance in GBM, although several questions about the mechanisms remain to be answered.

## MATERIALS AND METHODS

### Cell culture and treatment

U87, T98G, 293T and HeLa cells were cultured in Dulbecco's Modified Eagle Medium (DMEM) supplemented with 10% fetal bovine serum (FBS) and penicillin/streptomycin. HeLa cells were transfected using polyethylenimine. To generate stable cell lines, T98G and U87 cells were infected with viruses produced in 293T cells using lentiviral vectors for protein overexpression or knockdown.

U87 and T98G cells were treated with the HDAC8-specific inhibitor PCI34051 (Cayman Chemical Company) according to the indicated concentrations and times. Both U87 and T98G cells were treated with 250 µM temozolomide (TMZ) for 2 days.

### Plasmids

MGMT was subcloned into the PCMV3xFLAG vector. The cDNA was amplified using the FLAG MGMT EcoRI-F and FLAG MGMT BamHI-R primers (Supplementary Table S1) and using cDNA from T98G cells as a template. Then, it was subcloned to pCR8/GW/TOPO TA using the pCR8/GW/TOPO TA cloning kit from Life Technologies (K250020) and TA FLAGHDAC8-F and FLAGMGMTBamHI-R primers for fragment amplification, after which it was introduced into the pLenti CMV Neo DEST vector (Addgene plasmid # 17392) using Gateway LR clonase II enzyme mix from ThermoFisher.

The FLAG-HDAC8 vector, which was published previously [43], was subcloned into pLenti CMV Puro DEST (Addgene plasmid # 17452) with the same procedure as for FLAG MGMT, using TAFLAGHDAC8-F and TAFLAGHDAC8-R. HDAC8 was subcloned into pcDNA3.1 HA and pcDNA3.1/Myc-His (Invitrogen) vectors using HAHDAC8-EcoRIF and HAHDAC8-XhoIR primers and pcDNA3.1mycHisHDAC8XhoI-F and pcDNA3.1mycHisHDAC8KpnI-R primers, respectively, and the FLAG-HDAC8 vector as a template.

pcDNA5-Adrm1-Flag (plasmid # 19418) and pcDNA3 HA-UCH37 (plasmid #19415) were obtained from Addgene. ADRM1 was subcloned into the pcDNA3.1 HA vector using the primers HA-ADRM1 BamHI-Fw and HA-ADRM1XhoI-Rv and FLAG-ADRM1 vector as a template. The HA-ADRM1 was subcloned into pLenti CMV Neo DEST with the same procedure as for FLAG-MGMT using ADRM1TAcloningR and HAADRM1 TA cloning F primers. For the subcloning of the truncated forms, pcDNA5-ADRM1-FLAG vector was used and amplification was subsequently performed using deltaDEUBADADRM1EcoRI-F and deltaDEUBADADRM1BamHI-R primers for DEUBAD and deltaPruADRM1EcoRI-F and deltaPruADRM1BamHI-R primers for DPRU. The fragments were introduced into the PCMV3xFLAG vector.

The HDAC8 and ADRM1 shRNA vectors were purchased from SIGMA Mission (HDAC8: 1-TRCN: TRCN0000350469, 2-TRCN0000004852 and 3-TRCN0000314874; and ADRM1: 1-TRCN0000286432, 2-TRCN0000293817).

### Viability assay

For the cell viability assay, Cell Counting Kit 8 (CCK-8, Dojindo) was used following the manufacturer instructions.

### Protein extraction and western blotting

Protein extraction was performed using a modified Dignam method, as described previously [44, 45]. Briefly, a cytoplasmic extraction was made with buffer A (10 mM Tris pH 7.8; 10 mM KCl; 1.5 mM MgCl_2_) and a nucleoplasmic extraction was made with buffer C (10 mM Tris pH 7.8; 0.42 M NaCl; 1.5 mM MgCl_2_; 0.2 mM EDTA; 25% glycerol). Then, both fractions were mixed. For histone extraction, an acidic extraction of the pellet with 0.2M HCl was performed following protein isolation.

For the Western blotting procedure, proteins were separated on SDS-PAGE and transferred onto a nitrocellulose membrane. The membrane was blocked with 5% milk in PBS-0.1% Tween, incubated with a primary antibody diluted in PBS-0.1% Tween as directed by the antibody supplier (Supplementary Table 2), and subsequently incubated with HRP secondary antibody. The image was detected using the Odyssey FC LI·COR machine.

### RNA extraction and qPCR

mRNA was extracted from the cells using Trizol from Fisher Scientific (15596018), following manufacturer instructions. cDNA was obtained from the extracted mRNA using the qScript cDNA synthesis kit (Quanta Biosciences, 101414-112). Quantitative PCR was performed with iQ SYBR green Supermix (Bio-Rad, 1708882). The results were normalized with EEF2, HPRT1 and NCL2. Primer sequences are available in Supplementary Table S3.

### Immunoprecipitation

Protein extracts were incubated overnight with α-FLAG resin (Sigma-Aldrich A2220) or α-HA agarose resin (Sigma-Aldrich A2095). Beads were washed twice with BC100 buffer (10 mM Tris pH 7.8, 0.5 mM EDTA, 10% glycerol, 100 mM KCl) and three times with BC500 buffer (10 mM Tris pH 7.8, 0.5 mM EDTA, 10% glycerol, 500 mM KCl). Subsequently, proteins were eluted with 0.2 M Glycine pH 2.

### Mass spectrometry analysis

Samples were separated in a 4%-16% SDS-PAGE and the differential 45 KDa band was prepared for protein identification analysis. The analysis was performed by the Mass Spectrometry and Proteomics Resource Core at Harvard FAS Division of Science Core Facility. The band was enzymatically digested and run on a nano-capillary HPLC/MSMS.

### Immunofluorescence

Cells were plated on coverslips and treated before the procedure. Then, the cells were fixed in 4% paraformaldehyde for 10 min at room temperature and were permeabilized for 10 min with 0.1% Triton-X in PBS1x, after which they were blocked for 1 hour with 5% bovine serum albumin (BSA) in PBS1x. Primary and secondary antibodies were diluted in 0.1% Triton-X and 5% BSA in PBS1x. The primary antibodies used were γ-H2AX (Upstate 05-636), 53BP1 (Novus Biologicals NB100-304ss) and HDAC8 (Abcam ab187139), which were diluted 1:150. As secondary antibodies, anti-rabbit Alexa Fluor 488 and anti-mouse Alexa Fluor 568, from Molecular Probes, were used. Cell nuclei were stained with DAPI (4’,6-diamidino-2- phenylindole). The Olympus, IX-LWPO microscope was used for image capture.

### Comet assay

The Comet Assay kit from Trevigen (#4250-050-K) was used for the alkaline comet assay, following the instructions from the manufacturer, and Sybr Green was used for nuclei staining. It was visualized with the Echo laboratory microscope. Analysis was performed with ImageJ software.

### Flow cytometry analysis

For the cell cycle distribution analysis, cells were treated with PCI34051 for 24 h or infected with HDAC8 shRNA and selected with puromycin for 5 days. Then, approximately 106 cells were fixed with 70% ethanol overnight and incubated at 37°C for 30 minutes with a propidium iodide mixture (50 µg/ml PI and 100 µg/ml RNase A in PBS). DNA content was analyzed using the BD Celesta Cell Analyzer flow cytometer and FlowJo software.

### Statistical analysis

Statistical analysis was performed with Microsoft Excel software and was subjected to the analysis of variance (ANOVA). The statistical significance and the p-values are indicated in the graphics.

## SUPPLEMENTARY DATA FIGURES AND TABLES



## Abbreviations

GBM: glioblastoma
MGMT: O^6^-methylguanine-methyltranferase DNA repair enzyme
TMZ: temozolomide
ADRM1: Adhesion Regulating Molecule 1
UCH37: Ubiquitin-Carboxyl terminal Hydrolase 37
