# Supplementary material for: HDAC8 affects MGMT levels in glioblastoma cell lines via interaction with the proteasome receptor ADRM1
Source: Genes Cancer. 2019;10(5-6):119–33. doi: 10.18632/genesandcancer.197 (PMC6872666; doi:10.18632/genesandcancer.197)
Supplement: Supplementary file 1 [file ganc-10-119-s001.pdf]

SUPPLEMENTARY DATA

HDAC8 affects MGMT levels in glioblastoma cell lines via interaction with the proteasome receptor ADRM1

Irene Santos-Barriopedro[#], Yixuan Li, Sonali Bahl and Edward Seto*

From George Washington Cancer Center, Department of Biochemistry & Molecular Medicine, George Washington University School of Medicine & Health Sciences, Washington, DC 20037, USA

[#]Present address: Department of Molecular Biology, Radboud Institute for Molecular Life Sciences (RIMLS), Radboud University, Nijmegen 6525GA, The Netherlands

* To whom correspondence should be addressed. Tel: +1 202 994 3156; Email: seto@gwu.edu

Content

- Figures S1-S6
- Tables S1-S3

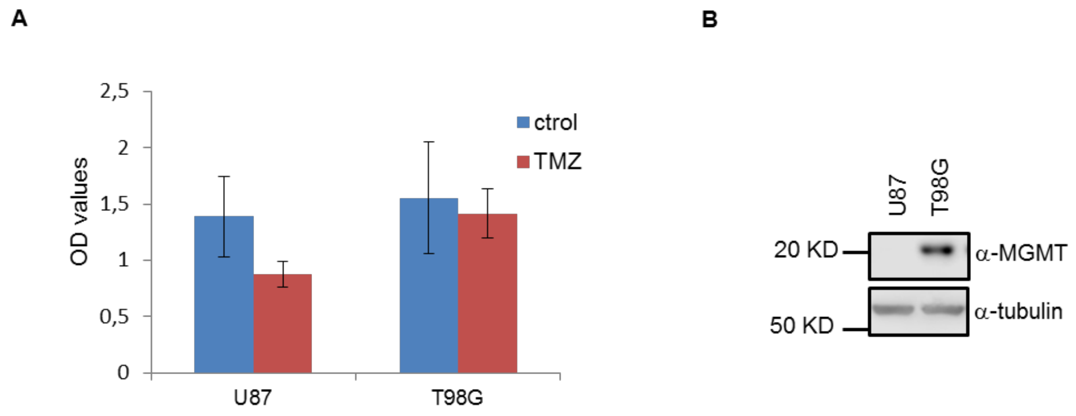

Figure S1. (A) Viability of U87 and T98G cells following treatment with TMZ for 3 days was assessed by the CCK-8 assay. **(B)** Extracts of U87 and T98G cells were subjected to Western blotting with MGMT and tubulin antibodies.

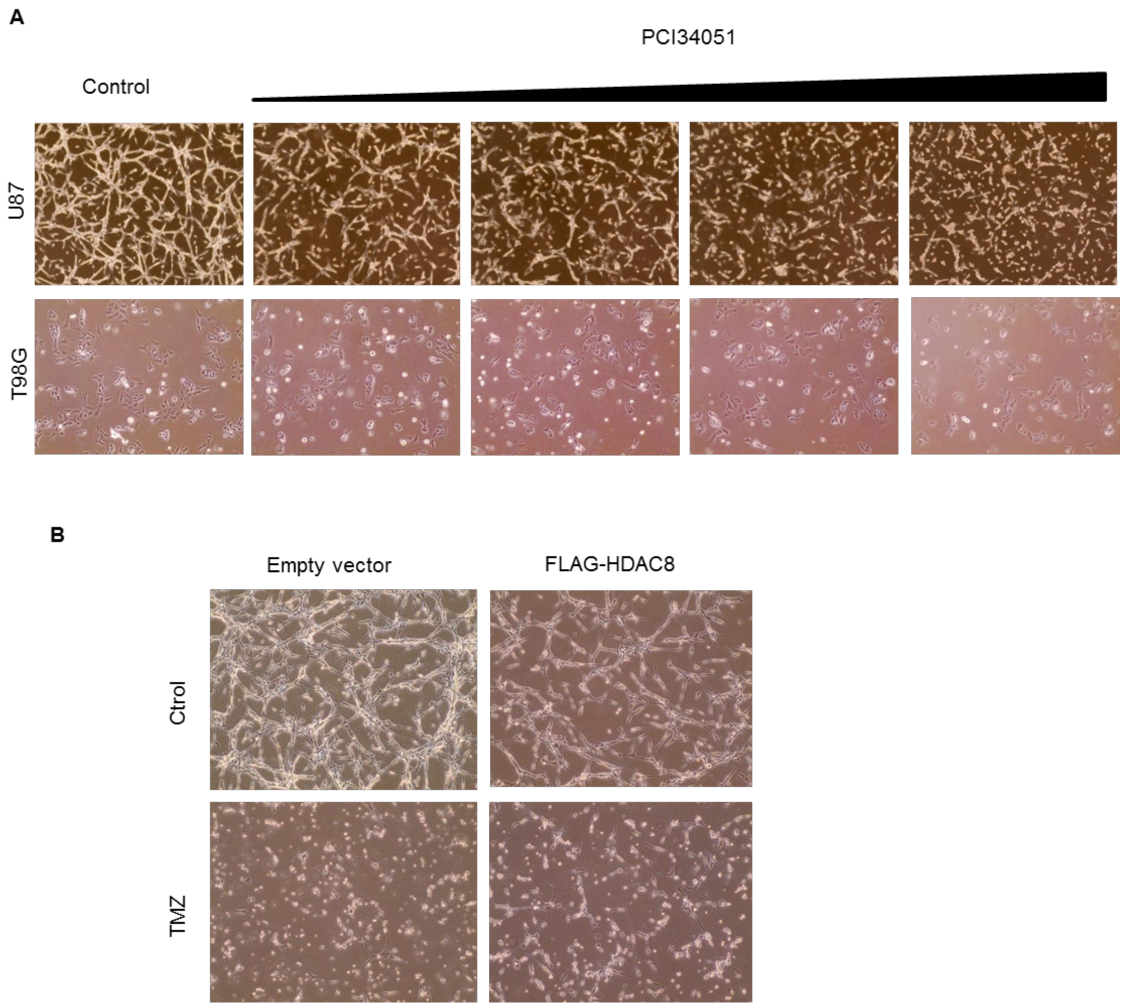

Figure S2. (A) Image capture of U87 and T98G cells after treatment with increasing concentrations of PCI34051 for 24 hours. (**B)** Image capture of U87 cells expressing FLAG-HDAC8 and treated with TMZ for 48 hours.

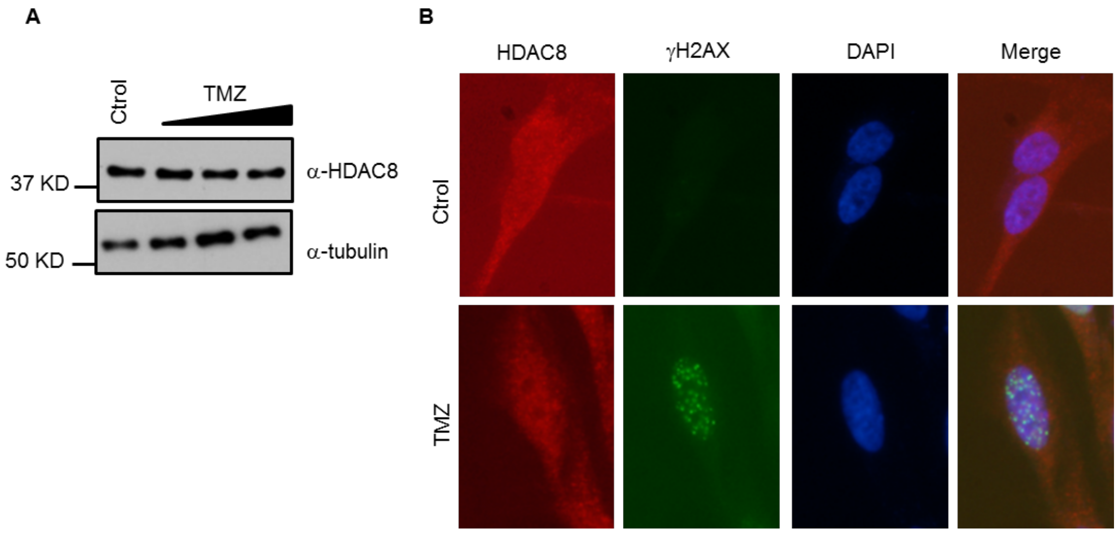

Figure S3. (A) Extracts from U87 cells treated with TMZ for 48 hours were subject to Western blotting with HDAC8 and tubulin antibodies. (**B)** Immunofluorescence to detect HDAC8 and γH2AX expression in U87 cells treated with TMZ for 48 hours.

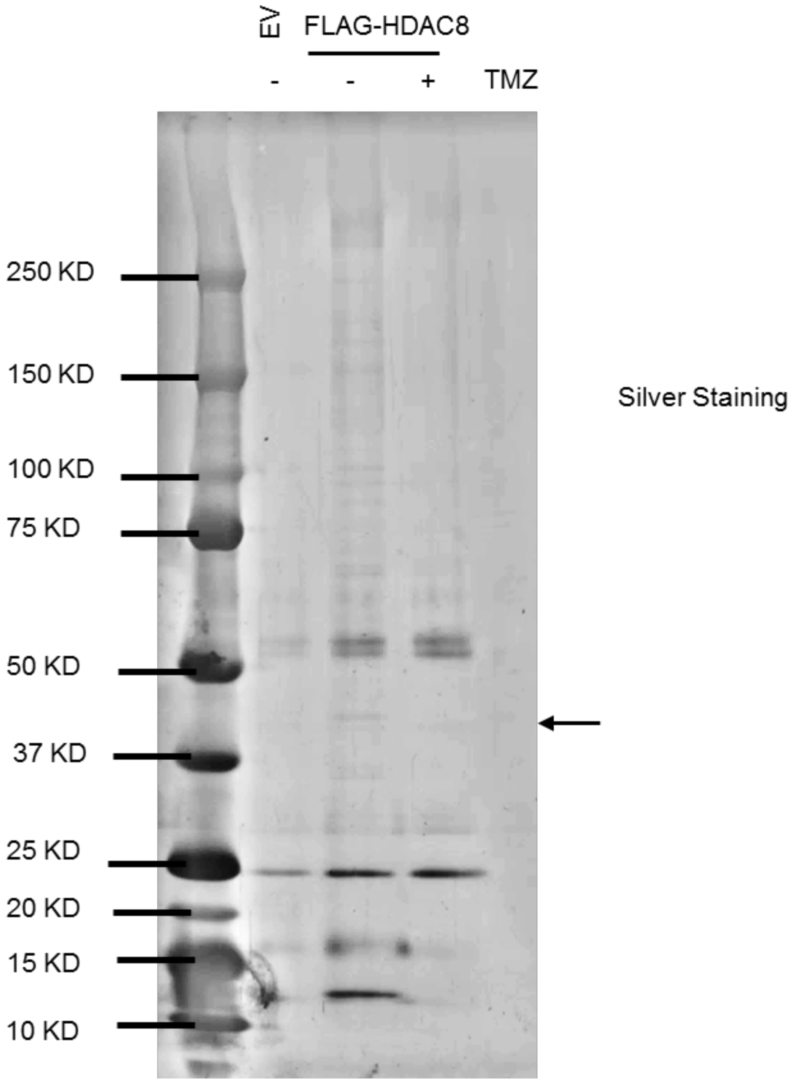

Figure S4. Extracts from U87 cells treated with TMZ for 48 hours were immunoprecipitated with a FLAG antibody and subject to Silver staining.

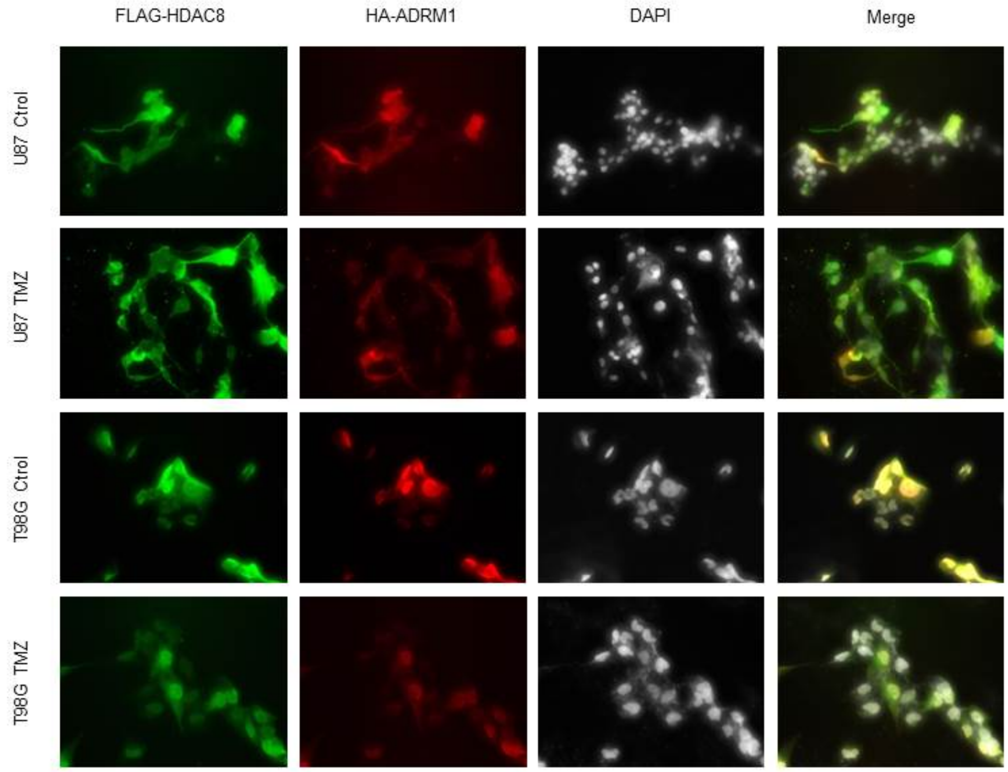

Figure S5. Fluorescence image of U87 and T98G cells expressing FLAG-HDAC8 and HA-ADRM1 and treated with TMZ for 48 hours.

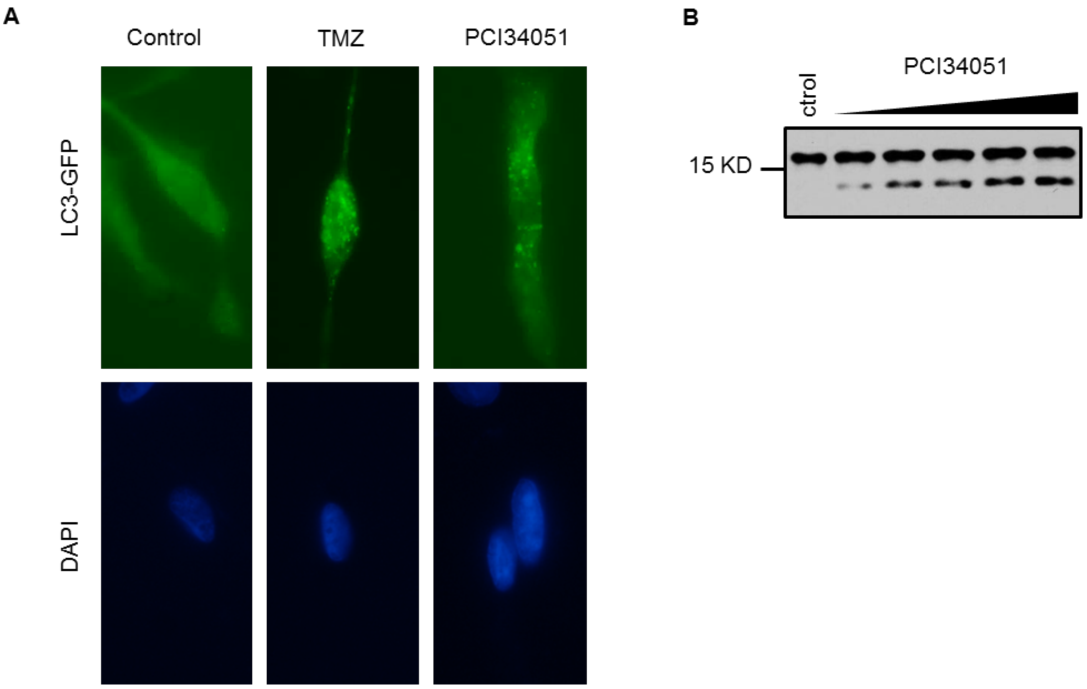

Figure S6. (A) Fluorescence image to visualize LC3-GFP expression in U87 cells treated with TMZ and PCI34051 for 24 hours. (**B)** Extracts of U87 cells treated with PCI34051 for 24 hours were subject to Western blotting with LC3 antibody.

Table S1. Primers used for subcloning

deltaDEUBADADRM1EcoRI-F	GGGAATTCGATGACGACCTCAGGCGCGCTCTTTC
deltaDEUBADADRM1BamHI-R	GGGGATCCTCACAGCTGGATGGCTGGGTCGGG
deltaPruADRM1EcoRI-F	GGGAATTCAGGCGGTGAGGGTGGCCTGCAGAGCC
deltaPruADRM1BamHI-R	GGGGATCCTCAGTCCAGGCTCATGTCCTC
FLAGMGMTEcoRI-F	GGGAATTCTATGCTGGGACAGCCCGCGCCCCT
FLAGMGMTBamHI-R	GGGGATCCTCAGTTTCGGCCAGCAGGCG
TAFLAGHDAC8-R	CTAGACCACATGCTTCAGATTCCCTTTG
TAFLAGHDAC8-F	ATGGACTACAAAGACCATGACGGTGATTAT
HAHDAC8-EcoRIF	CCGGAATTCATGGAGGAGCCGGAGGAACCGGCGGAC
HAHDAC8-XhoIR	CCGCTCGAGCTAGACCACATGCTTCAGATTCCCTTTG
HA-ADRM1 BamHI-Fw	CCGGATCCATGACGACCTCAGGCGCGCTCTTTC
HA-ADRM1XhoI-Rv	GGCTCGAGTCAGTCCAGGCTCATCTCC
ADRM1TAcloningR	TCAGTCCAGGCTCATGTCCTC
HAADRM1 TA cloning F	ATGGACTACCCTTATGATGTGCCGG
pcDNA3.1mycHisHDAC8XhoI-F	CCCTCGAGATGGAGGAGCCGGAGGAACCGGCG
pcDNA3.1mycHisHDAC8KpnI-R	CCGGTACCGACCACATGCTTCAGATTCCC

Table S2. List of antibodies used for Western blot

Protein targeted	Company	Cat. No.	Dilution
HA	Sigma	H9658	1:1000
FLAG	Sigma	F3165	1:1000
tubulin	DSH	AB_1157911	1:5000
vinculin	Santa Cruz	sc-73614	1:1000
actin	Sigma	A1978	1:5000
HDAC8	Abcam	Ab187139	1:5000
ADRM1	CST	12019S	1:1000
MGMT	Santa Cruz	sc-166516	1:200
PARP	CST	9532	1:1000
Phosphor Cdc2 Y15	CST	4539	1:1000
phosphoH3S10	CST	3377	1:1000
Cyclin A	CST	4656	1:1000
γ-H2AX	Upstate	05-636	1:1000
H3	CST	9715	1:1000
Phosphor ATM S1981	CST	4526	1:1000
ATM	Genetex	GTX70103	1:1000
PSMD1	Bethyl	A303-852A-T	1:1000
myc	CST	2276	1:1000

Table S3. Primers used for qPCR

MGMT-F	AGCTGATGCCGTGGAGGT
MGMT-R	ACGACTCTTGCTGGAAAACG
hEEF2 F	TGGAGATCTGCCTGAAGGAC
hEEF2 R	GACTTGGAGAGGCAGAGAGCAC
hNCL-F	CTGCCTCAGAGGATGAG
hNCL-R	TCTGTTTGGCCATTTCCTTC
hHPRT1-F	TGACACTGGCAAAACAATGCA
hHPRT1-R	GGTCCTTTTCACCAGCAAGCT
HDAC8-F	GCGTGATTTCCAGCACATAA
HDAC8-R	ATACTTGACCGGGGTCATCC
